# Structure and function of retinal ganglion cells in subjects with a history of repeated traumatic brain injury

**DOI:** 10.3389/fneur.2022.963587

**Published:** 2022-08-12

**Authors:** Kelly R. Klimo, Elizabeth A. Stern-Green, Erica Shelton, Elizabeth Day, Lisa Jordan, Matthew Robich, Julie Racine, Catherine E. McDaniel, Dean A. VanNasdale, Phillip T. Yuhas

**Affiliations:** ^1^College of Optometry, The Ohio State University, Columbus, OH, United States; ^2^Department of Ophthalmology, Nationwide Children's Hospital, Columbus, OH, United States

**Keywords:** traumatic brain injury, retinal ganglion cells, retinal nerve fiber layer, electroretinography, photopic negative response, optical coherence tomography, static automated perimetry, scanning laser polarimetry

## Abstract

This study tested whether repeated traumatic brain injuries (TBIs) alter the objective structure or the objective function of retinal ganglion cells (RGCs) in human subjects recruited from an optometry clinic. Case subjects (*n* = 25) with a history of repeated TBIs (4.12 ± 2.76 TBIs over 0–41 years) and healthy pair-matched control subjects (*n* = 30) were prospectively recruited. Retinal nerve fiber layer (RNFL) thickness was quantified with spectral-domain optical coherence tomography, and scanning laser polarimetry measured RNFL phase retardation. Measurements of the photopic negative response were made using full-field flash electroretinography. There was no statistically significant difference (*p* = 0.42) in global RNFL thickness between the case cohort (96.6 ± 9.4 microns) and the control cohort (94.9 ± 7.0 microns). There was no statistically significant difference (*p* = 0.80) in global RNFL phase retardation between the case cohort (57.9 ± 5.7 nm) and the control cohort (58.2 ± 4.6 nm). There were no statistically significant differences in the peak time (*p* = 0.95) of the PhNR or in the amplitude (*p* = 0.11) of the PhNR between the case cohort (69.9 ± 6.9 ms and 24.1 ± 5.1 μV, respectively) and the control cohort (70.1 ± 8.9 ms and 27.8 ± 9.1 μV, respectively). However, PhNR amplitude was more variable (*p* < 0.025) in the control cohort than in the case cohort. Within the case cohort, there was a strong positive (*r* = 0.53), but not statistically significant (*p* = 0.02), association between time since last TBI and PhNR amplitude. There was also a modest positive (*r* = 0.45), but not statistically significant (*p* = 0.04), association between time since first TBI and PhNR amplitude. Our results suggest that there were no statistically significant differences in the objective structure or in the objective function of RGCs between the case cohort and the control cohort. Future large, longitudinal studies will be necessary to confirm our negative results and to more fully investigate the potential interaction between PhNR amplitude and time since first or last TBI.

## Introduction

Traumatic brain injury (TBI) is a disruption in the normal function of the brain caused by a bump, blow, or jolt to the head or by a penetrating head injury (1). The rotational and acceleration-deceleration forces associated with these insults can impair axoplasmic transport and can induce axonal swelling in brain neurons through a process called diffuse axonal injury (2). In the United States, over 1.5 million people sustain a TBI each year (3). Having a previous TBI is a strong predictor for having another (4). It is therefore unsurprising that 35% of TBIs in athletes follow a previous TBI (5); that is, they are repeat injuries. Repeated TBIs can lead to a distinct pathophysiology, called chronic traumatic encephalopathy, where tau protein within the central nervous system becomes phosphorylated, leading to the degradation of axonal microtubules and to eventual neuronal death (6).

The diagnosis of and monitoring of TBI is a challenge for clinicians and for patients. In the clinic, TBI manifests as periods of decreased consciousness, amnesia, neurologic deficits, and alteration in mental state after the injury (7). Often, diagnosis and classification of TBI involves taking a detailed case history and assessing these signs and symptoms using batteries, such as the Glasgow Coma Scale (8) or the Sport Concussion Assessment Tool (9). This subjective approach is likely insensitive to mild or moderate TBI (10, 11), but it is necessary due to a lack of objective clinical markers of TBI pathology. Although imaging studies of the brain can detect pathology associated with severe TBI, such as intracranial hemorrhage (12), microstructural axonal injuries associated with mild or moderate TBIs may not be detected by computerized tomography (13) and are not relatable to a specific cause when detected with advanced imaging techniques, such as diffusion-tensor imaging (14). This lack of clinical markers for TBI likely prevents or delays diagnosis, which in turn diminishes the likelihood of a timely referral to the appropriate rehabilitation services and complicates return-to-work, return-to-school, and return-to-play decisions.

The neural retina may be a unique site to objectively detect TBI pathology. It arises from diencephalic neural ectoderm and shares the vascularization patterns of the brain. The long axons of retinal ganglion cells (RGCs) course through the brain to the lateral geniculate nucleus in the thalamus and to other targets and thus may be susceptible to the shearing forces of a TBI (15–17). Even if RGC axons escape direct mechanical insult, they may be vulnerable to TBI pathology through transsynaptic degeneration, where neurodegeneration in one region of the brain spreads to other regions, possibly through oxidative injury or glutamate excitotoxicity (18–20). There is evidence in mouse models that both a single TBI (15, 21–23) and repeated TBIs (24–26) can induce inflammation in the posterior segment, can reduce the density of RGCs, and can thin the retinal nerve fiber layer (RNFL). Functional impairment followed structural loss in these studies, as TBI diminished the contributions of RGCs both to the pattern electroretinography (ERG) waveform (21) and to the flash ERG waveform (24).

The results from animal models of TBI are starting to be replicated in human subjects. Clinical studies conducted on populations prone to frequent TBIs, such as athletes (27) and soldiers (28), have reported both thinning (29–31) and thickening (30, 32) of the neural retina as a result of TBIs. Thinning of the RNFL after TBI may be associated with the loss of visual field sensitivity (33); however, ERG testing has not elicited deficits in the outer retina in patients with TBI (34). The objective function of RGCs after a TBI remains unelucidated. It is also unclear whether repeated TBIs can alter the structure and the function of RGCs in a general population of non-soldiers and non-athletes. Thus, the purpose of this study was to test the hypothesis that repeated TBIs alter the objective structure or the objective function of RGCs in human subjects recruited from an optometry clinic.

## Materials and methods

This cross-sectional pilot study followed the tenants of the Declaration of Helsinki and was approved by the Institutional Review Board in Biomedical Sciences at The Ohio State University (OSU). Subjects provided informed consent to participate prior to data collection at the OSU College of Optometry. Sample size determination was based on reports of TBI-induced changes to retinal structure in athletes (30) and in veterans (29, 35) and on an attenuated photopic negative response (PhNR) amplitude in multiple sclerosis patients without a history of optic neuropathy (36).

### Subject recruitment and screening

Two subject cohorts were prospectively recruited from the OSU optometry clinics and from the university community. The first cohort comprised case subjects, who had a history of multiple mild or moderate TBIs, as defined by the Veterans Affairs/Department of Defense Clinical Practice Guideline for Management of Concussion/Mild Traumatic Brain Injury (37). Individuals with a history of severe TBI—defined as loss of consciousness of >24 h, alteration of consciousness or mental state for >24 h, posttraumatic amnesia of >7 days, or a Glascow Coma Score of <9—were not enrolled in the study. The second cohort comprised healthy control subjects.

All potential subjects were screened for initial eligibility criteria. Potential case participants were invited to schedule their first study visit if they: (1) were 18 years of age or older; (2) self-reported at least two mild-moderate TBIs; (3) did not self-report any eye diseases or conditions, save for refractive error; (4) did not self-report any neurological diseases, apart from TBI; (5) did not self-report diabetes; and (6) were tobacco non-users. Potential control participants were subject to the same screening questions, save they could not have a history of TBI. The controls were age- and sex-matched to the cases.

### Entrance testing

#### Assessment of traumatic brain injuries

For all participants, the study contained two sessions, separated by at least 24 h. At the beginning of the first session, the screening eligibility questions were reassessed, and a verbal administration of the OSU Traumatic Brain Injury Identification Method (OSU TBI-ID) was used to quantify TBI history. The OSU TBI-ID is a validated survey that is a reliable indicator of lifetime TBI history (38–40). To ensure an accurate TBI history, the results of the OSU TBI-ID were collaborated with the TBI history contained within the subject's OSU optometry chart, if available. Any discrepancies were resolved before the study session proceeded.

#### Physical examination

An optometrist (ES, ED, or PTY) then examined the anterior and posterior segments of the eyes. First, monocular distance visual acuities were acquired through habitual refractive error correction. Then, intraocular pressures were measured using Goldmann applanation tonometry, and both eyes were dilated using 1.0% tropicamide. Slit lamp biomicroscopy of the anterior segment and fundoscopy and binocular indirect ophthalmoscopy of the posterior segment were performed once the subject was dilated.

#### Eligibility assessment

Subject eligibility was reassessed after the physical examination. Case subjects were allowed to continue in the study if they: (1) had at least two lifetime mild-moderate TBIs, per the OSU TBI-ID; (2) had no lifetime severe TBIs, per the OSU TBI-ID; (3) were free from diabetes mellitus and neurological diseases outside of TBI, as confirmed by chart review; (4) had a corrected visual acuity of 20/30 or better in each eye; (5) had a maximum intraocular pressure of ≤21 mmHg in each eye; (6) manifested no posterior segment diseases that could impair retinal structure or function; and (7) manifested no anterior segment diseases that could obfuscate assessment of the retina. Control subjects were eligible to continue if they met the same criteria, with the exception that they could have no lifetime TBIs of any severity.

### Retinal nerve fiber layer imaging

**Figure 1:**
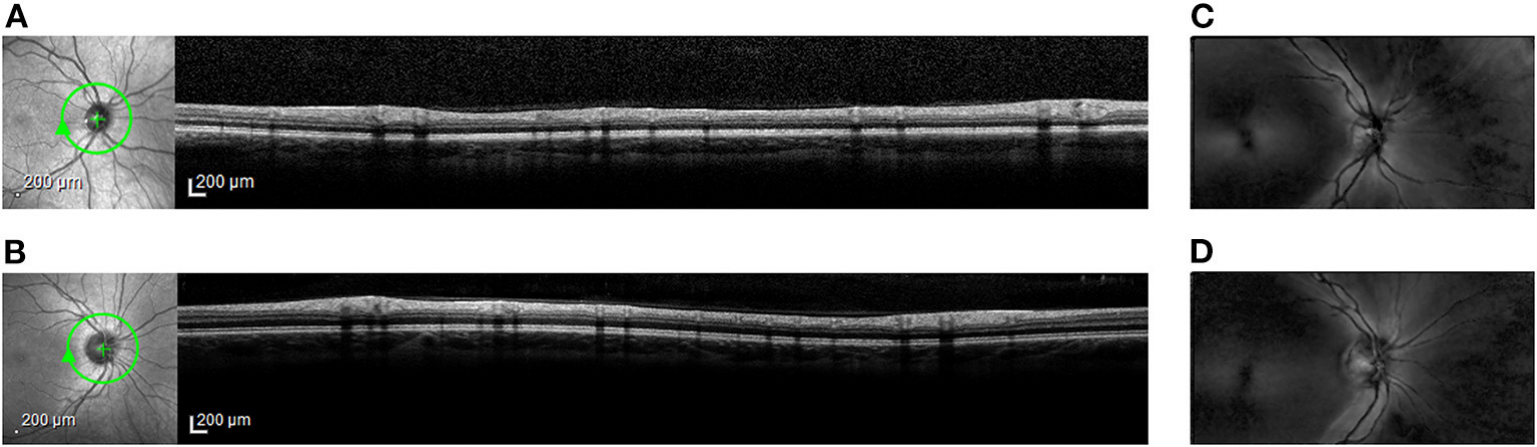
Representative retinal images from spectral-domain optical coherence tomography (OCT) and from scanning laser polarimetry (SLP). *En face* scanning laser ophthalmoscope (SLO) images (left) and cross-sectional OCT images (right) from **(A)** Case Subject 110 and from **(B)** Control Subject 203. The green circle around the optic nerve head in the SLO image marks the location of the accompanying OCT image. *En face* SLP images from **(C)** Case Subject 110 and from **(D)** Control Subject 203. Bright regions in the SLP image indicate areas of high phase retardation.

Subjects continuing in the study then sat for two retinal imaging procedures. First, spectral-domain optical coherence tomography (SD-OCT) images were taken of the retinal nerve fiber layer (RNFL; Figures 1A,B) by one of five study team members (KRK, EAS-G, ES, ED, or PTY) using commercial software (Eye Explorer, Version 1.10.0.0) on a single Spectralis instrument (Heielberg Engineering; Heidelberg, Germany). Specifically, a 12° (~3.6 mm in diameter) circular scan manually centered on the optic nerve head was acquired for each subject. To minimize noise, each image used for analysis was the composite average of 100 individual registered b-scans acquired using Heidelberg TruTrack Active Eye Tracking. Images were acquired at the high-resolution setting, which collects 1,536 a-scans per b-scan. The nominal axial and lateral resolutions were 7 and 14 μm, respectively. The infrared beam of the scanning super luminescence diode had an average wavelength of 870 nm.

Second, scanning laser polarimetry (SLP) with variable corneal compensation was used to measure the phase retardation caused by RNFL in the peripapillary retina by one of five study team members (KRK, EAS-G, ES, ED, or PTY) using commercial software (Version 5.5.0.11) on a single GDx VCC instrument (Carl Zeiss Meditec; Jena, Germany). Specifically, a 20° × 40° macular-centered image (Figures 1C,D) was collected from both eyes of each subject using a raster-scanning 780 nm polarized light source focused on the retina. Three serial scans were obtained with each test.

#### Image processing and analysis

RNFL structural measurements were made from the OCT and SLP images. The RNFL was automatically segmented in Eye Explorer. Global RNFL thickness values and localized RNFL thickness measurements (temporal, temporal-superior, temporal-nasal, nasal, nasal-superior, and nasal-infection sectors) were recorded. For the SLP, RNFL phase retardation was determined along a 3.2-mm-diameter circle, automatically centered on the disc. Global RNFL phase retardation values were calculated from the SLP images by the device's internal software and then recorded. Localized RNFL phase retardation values were also recorded from the superior and inferior quadrants.

All images were inspected for quality, and images with movement artifacts, fixation anomalies, segmentation errors, misaligned measurement beams, or poor quality scores (<20 dB for OCT images and <8 for the SLP images) were removed from analysis. For both OCT and SLP, data were recorded from both eyes and were averaged for an aggregate value for each subject. If data from both eyes were unavailable, data from one eye were used.

#### Statistical analysis

Global RNFL thickness and global RNFL phase retardation were the primary outcome measures of OCT imaging and of SLP imaging, respectively. Two-tailed paired-sample *t*-tests (statistical significance cutoff α = 0.05) compared these indices between the case cohort and the control cohort. Secondary outcome measures were also considered. For OCT imaging, sectoral RNFL thicknesses were compared between the two cohorts. For SLP imaging, superior and inferior quadrant RNFL phase retardation values were compared between the two cohorts. To account for the large number of multiple comparisons, the cutoff for statistical significance was adjusted to α = 0.01 for the secondary analysis tests. This statistical significance level was chosen to control the type I error rate while avoiding inflation of type II error.

F-tests for equal variances analyzed differences in variance between the case cohort and the control cohort for the primary imaging measures. If the f-statistic value was greater than f-critical value for any measure, the variances for that measure in the case cohort and the control cohort were not equal at the statistical significance level α = 0.025, to account for multiple comparisons.

### Perimetry and electroretinography recordings

At the beginning of the second study session all participants were asked about changes to their medical, ocular, and TBI histories. Monocular distance visual acuities were measured through habitual refractive error correction. 1.0% tropicamide was then instilled into both eyes. While the eyes dilated, a white-on-white static 30-2 threshold visual field was obtained from each eye by one of five study team members (KRK, EAS-G, ES, ED, or PTY) using a single Octopus 600 perimeter (Hagg-Streit; Koniz, Switzerland; TOP testing strategy) or a single Humphrey Field Analyzer 3 perimeter (Carl Ziess Meditec; SITA-FAST testing strategy). Mean deviation (MD) and pattern standard deviation (PSD) values were recorded from visual fields that were free from artifacts (e.g., trial lens scotoma), had fewer than 33% fixation losses, and had false positive and false negative rates of <20%.

Once fully dilated, subjects were prepared for full-field flash electroretinography (ERG) using a Veris Pro 6.4.5 instrument (Electro-Diagnostic Imaging; Milpitas, CA) by one of four study team members (KRK, EAS-G, ES, or ED). Specifically, topical anesthetic (proparacaine 0.5%) was first instilled into each eye. Dawson, Trick, and Litzkow (DTL) Plus electrodes (Diagnosys; Lowell, MA) were then placed deep inside the lower conjunctival fornix of each eye. The DTL Plus electrodes were referenced to skin electrodes placed near the ipsilateral temporal canthus of each eye, and a ground electrode was positioned at the center of the forehead. Participants were then positioned in front of the device's Ganzfeld dome for 12 bilateral ERG recordings of the PhNR in accordance with the International Society for Clinical Electrophysiology of Vision (41, 42).

#### Waveform processing and analysis

**Figure 2:**
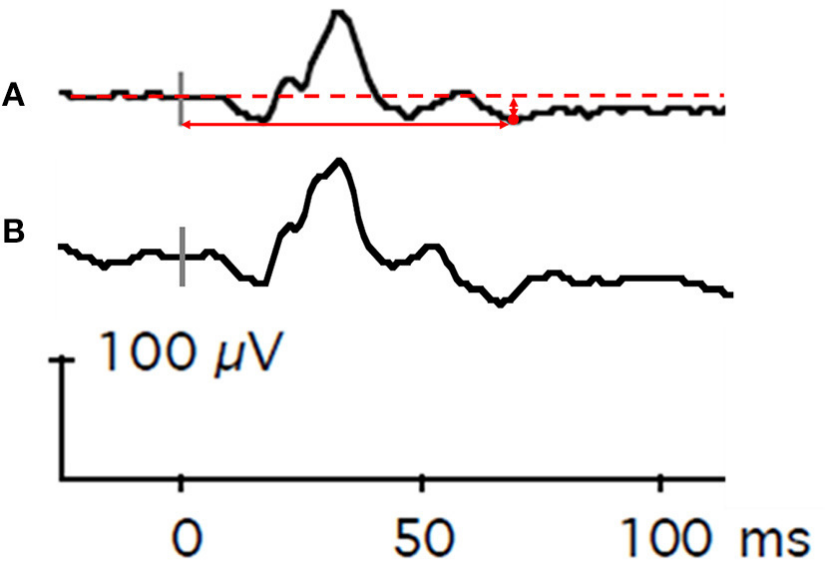
Representative flash electroretinography waveforms. **(A)** This composite waveform was generated from Case Subject 101 by averaging 12 individual waveforms elicited by repeated presentations of a red light (4 ms duration, 3.05 cd•s/m^2^) against a static blue background (10.80 cd•s/m^2^). The dashed horizontal red line is baseline voltage. The vertical gray bar indicates light onset. The red circle denotes the lowest voltage of the waveform after the B-wave. The vertical solid red arrow represents PhNR amplitude, and the horizontal solid red arrow represents PhNR peak time. **(B)** For comparison, a composite waveform generated from Control Subject 202. The vertical gray bar indicates light onset.

Measurement of the PhNR from ERG recordings was used to assess the objective function of RGCs (43, 44). For each eye, the 12 individual recordings were visually inspected for quality, and recordings with artifacts from eye movements or from blinks were removed from analysis. The remaining individual waveforms were then averaged in each eye. PhNR was manually identified by one study team member (KRK) on the averaged waveform as the nadir occurring after the B-wave (Figures 2A,B). The PhNR amplitude was measured from baseline, and the PhNR peak time was measured from light onset. Outlying amplitude values and peak time values, defined as being outside of ±2 standard deviations from the mean value of each eye, were removed from analysis. Finally, amplitude and peak time values from each eye were averaged for aggregate amplitude and peak time values, respectively, for each subject. If data from both eyes were unavailable, data from one eye were used.

#### Statistical analysis

PhNR amplitude and PhNR peak time were the primary outcome measures of the ERG recordings and of objective RGC function. MD and PSD from perimetry were secondary outcome measures for RGC function. Two-tailed paired-sample *t*-tests (statistical significance cutoff α = 0.05) compared these parameters between the case cohort and the control cohort. F-tests for equal variances analyzed differences in variance between the case cohort and the control cohort for the primary ERG measures. If the f-statistic value was greater than f-critical value for any measure, the variances for that measure in the case cohort and the control cohort were not equal at the statistical significance level α = 0.025, to account for multiple comparisons.

### Analysis of structure and function associations

Linear structure-function relationships between the primary imaging outcome measures and the primary ERG outcome measures were assessed for the case cohort using Pearson correlation coefficient tests performed on the continuous data (statistical significance cutoff α = 0.01, to account for multiple comparisons). Specifically, correlations were made between global RNFL thickness and PhNR amplitude, global RNFL thickness and PhNR peak time, global RNFL phase retardation and PhNR amplitude, and global RNFL phase retardation and PhNR peak time. The strength of correlations was considered weak for coefficients ±0.1–0.3, moderate for coefficients ±0.3–0.5, and strong for coefficients ±0.5–1.0 (45).

#### Associations between traumatic brain injury history and retinal structure and function

Associations between TBI history and the primary outcome measures of the imaging and of the ERG tests were assessed using the Spearman's Rank correlation coefficients tests performed on the continuous data (statistical significance cutoff α = 0.01, to account for multiple comparisons). Specifically, the relationships between number of lifetime TBIs and primary outcome measures, between time since last TBI and primary outcome measures, and between time since first TBI and primary outcome measures were analyzed. The strength of correlations was assessed using the scale provided above.

Case subjects were also categorized to illustrate their TBI histories. For example, case subjects were divided into three subgroups (2–3 TBIs, 4–5 TBIs, and >5 TBIs) to demonstrate the effect that number of TBIs had on the primary outcome measures. Similarly, cases subjects were divided into three subgroups (<2, 2–4, and ≥5 years) to demonstrate the effect that time since their last TBI had on the primary outcome measures. Finally, cases subjects were divided into four subgroups (≤5, 6–10, 11–20, and >20 years) to demonstrate the effect that time since their first TBI had on the primary outcome measures. These categorizes were chosen to evenly distribute the sample population, and they were not the basis of any correlation testing.

As a secondary analysis, associations between TBI history and the perimetry outcome metrics (e.g., MD and PSD, values averaged between the two eyes) were assessed using the Spearman's Rank correlation coefficients tests performed on the continuous data (statistical significance cutoff α = 0.01, to account for multiple comparisons). The strength of correlations was assessed using the scale provided above.

## Results

### Study participants

Twenty-five (*n* = 25) case subjects [mean ± standard deviation (SD) age = 32.2 ± 11.8 years; 52% female] were enrolled in the study. All case subjects completed the first study session, and all but two returned for the second study session. Case subjects reported an average (± SD) of 4.12 ± 2.76 TBIs over a range of 0–41 years prior (Table 1).

**Table 1:** Traumatic brain injury (TBI) history of case subjects.

**Subject**	**Number of TBIs (OSU TBI-ID)**	**Causes (and numbers) of TBIs**	**Years since first TBI**	**Years since last TBI**
101	2	Fall (2)	1	1
102	2	MVA (2)	3	1
103^a^	5	Strike to head (3), Fall (1), MVA (1)	5	1
104	2	Strike to head (2)	11	10
105	6	Strike to head (4), Fall (1), MVA (1)	7	2
106	3	Athletics (1), Fall (1), MVA (1)	18	8
107	6	Fall (5), MVA (1)	18	3
108	3	Athletics (3)	8	2
109	3	Fall (2), Strike to head (1)	9	4
110	2	Fall (1), MVA (1)	6	2
111	5	Athletics (3), Assault (1), MVA (1)	9	2
112	2	Athletics (2)	7	5
113	3	Strike to head (2), MVA (1)	7	0
114^a^	15	Assault (6), Strike to head (5), Athletics (3), Fall (1)	9	0
115	3	Fall (2), MVA (1)	4	1
116	4	Fall (2), MVA (2)	34	2
117	2	Athletics (1), Strike to head (1)	29	18
118	6	Athletics (6)	40	2
119	4	Blast (2), Fall (1), MVA (1)	17	12
120	3	Athletics (2), Strike to head (1)	17	1
121	4	Athletics (3), MVA (1)	15	4
122	8	MVA (3), Assault (2), Athletics (2), Fall (1)	41	5
123	3	Athletics (3)	13	7
124	3	Athletics (2), Assault (1)	2	1
125	4	Fall (2), Assault (1), MVA (1)	11	1

a These two subjects completed the first study visit but did not return for the second study visit. MVA, motor vehicle accident; OSU TBI-ID, Ohio State University Traumatic Brain Injury Identification Method.

Thirty (*n* = 30) age- and sex-matched control subjects (age = 34.4 ± 12.6 years; 47% female) were enrolled in the study. The OSU TBI-ID identified TBIs in four of the enrolled control subjects, and optic nerve head drusen were discovered in one control subject during the dilated fundus examination. These five control subjects were dismissed from the study before retinal imaging and before ERG testing. One control subject completed the first study session but did not return for the second session. Table 2 contains the results of the ophthalmic examinations of the case subjects and of the control subjects and their self-reported races. There were no statistically significant differences between the cohorts in race, intraocular pressures, cup-to-disc ratios, mean deviations, and pattern standard deviations. Control subjects were statically significantly more myopic in both eyes than case subjects, however. These clinically modest differences in refractive error likely did not affect the retinal imaging results (46) or the ERG results (47).

**Table 2:** Characteristics of all study participants.

	**Case cohort**	**Control cohort**	***P*-value**
**Race**			0.90
White	25 (100%)	29 (97%)	
Black	0 (0%)	1 (3%)	
**Refractive error (spherical equivalent diopters)**			
OD	−0.95 ± 1.62	−2.58 ± 1.90	0.01^a^
OS	−0.90 ± 1.77	−2.74 ± 1.90	0.01^a^
**Intraocular pressure (mmHg)**			
OD	15.1 ± 2.72	14.8 ± 2.75	0.83
OS	15.1 ± 2.47	14.8 ± 2.72	0.78
**Cup-to-disc ratio**			
OD	0.31 ± 0.10	0.32 ± 0.09	0.71
OS	0.31 ± 0.10	0.33 ± 0.11	0.56
**Visual field mean deviation (dB)**			
OD	−1.47 ± 3.61	−1.27 ± 2.10	0.85
OS	−1.19 ± 2.44	−0.88 ± 1.56	0.92
**Visual field pattern standard deviation (dB)**			
OD	1.80 ± 0.23	1.72 ± 0.16	0.45
OS	1.76 ± 0.22	1.62 ± 0.11	0.51

Values are mean ± standard deviation, except for race, which is number of subjects (percent of cohort).

a Statistically significant difference (p < 0.05, paired t-test) between the case cohort and the control cohort. OD, right eye; OS, left eye.

### Retinal nerve fiber layer imaging

**Figure 3:**
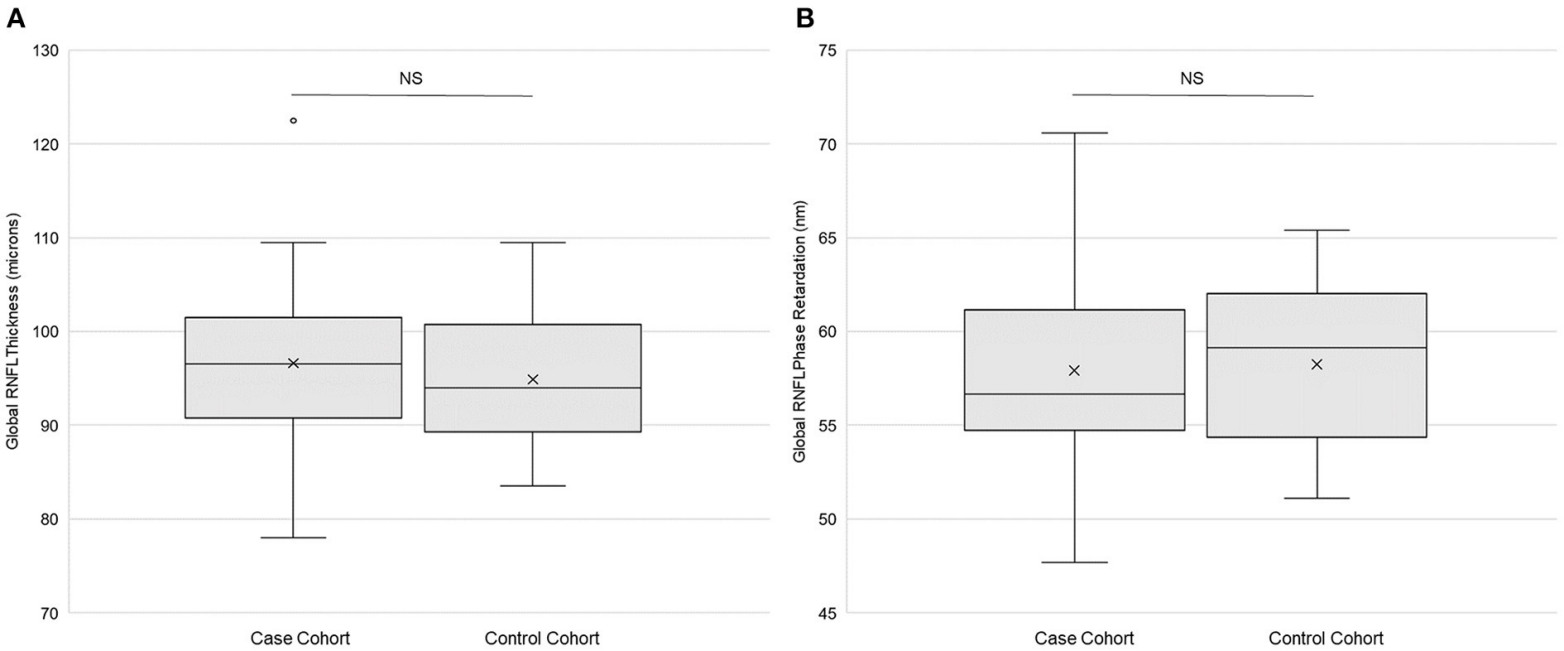
Retinal imaging primary outcomes. **(A)** Global retinal neve fiber layer (RNFL) thickness (*n* = 25) and **(B)** global RNFL phase retardation (*n* = 24) in both the case and the control cohorts. Each box represents the interquartile range, and the internal line is the median. The internal “X” is the mean. The whiskers represent the 90th and 10th percentiles, and the filled circle is an outlying value. Phase retardation data for one case subject (114) were not collected due to a technical difficulty. NS is not statistically significant (*p* > 0.05, paired *t*-test).

There were no statistically significant differences in global RNFL thickness (*p* = 0.42, paired *t*-test; Figure 3A) or in global RNFL phase retardation (*p* = 0.80; Figure 3B) between the case cohort and the control cohort. Likewise, a comparison of the ratio of global RNFL phase retardation to global RNFL thickness did not reveal a statistically significant difference (*p* = 0.24) between the case cohort (mean ± SD = 0.60 ± 0.04 nm) and the control cohort (0.62 ± 0.05 nm). Variance in the case cohort was not statistically significantly different (*p* > 0.025, *f*-test for equal variances) from variance in the control cohort, both for global RNFL thickness and for global RNFL phase retardation (Table 3). Supplementary Table 1 presents the primary outcomes of the retinal imaging tests according to sex. There were also no statistically significant differences between the two cohorts in sectoral RNFL thicknesses or in quadrantile phase retardations (Table 4).

**Table 3:** Inter-cohort comparison of the variance of the primary imaging and electroretinography outcome measures.

	**Direction**	**Variance ratio**	**F-critical**	**F-statistic**
Global RNFL thickness (*n* = 25)	$\frac{Variace\left(Case\right)}{Variance\left(Control\right)}$	$\frac{89.0}{49.4}$	2.27	1.80
Global RNFL phase retardation (*n* = 25)	$\frac{Variace\left(Case\right)}{Variance\left(Control\right)}$	$\frac{32.2}{21.5}$	2.31	1.50
PhNR amplitude (*n* = 20)	$\frac{Variace\left(Control\right)}{Variance\left(Case\right)}$	$\frac{83.5}{26.4}$	2.53	3.17^a^
PhNR peak time (*n* = 21)	$\frac{Variace\left(Control\right)}{Variance\left(Case\right)}$	$\frac{79.2}{47.8}$	2.46	1.66

a Statistically significant difference in variance (p < 0.025, f-test for equal variances) between the case cohort and the control cohort. RNFL, retinal nerve fiber layer; PhNR, photopic negative response.

**Table 4:** Secondary retinal nerve fiber layer (RNFL) imaging outcomes.

	**Case**	**Control**	**Mean**	***P*-value**
	**cohort**	**cohort**	**difference**	
**Sectoral RNFL thickness (μm**, ***n*** **=** **25)**				
Temporal	67.8 ± 9.4	72.9 ± 11.2	−5.1 ± 14.6	0.093
Superior-temporal	131.2 ± 16.7	130.0 ± 12.4	2.2 ± 21.3	0.608
Superior-nasal	104.0 ± 19.5	98.2 ± 21.8	5.8 ± 32.0	0.375
Nasal	74.5 ± 12.1	67.1 ± 9.9	7.4 ± 13.4	0.011
Inferior-nasal	111.9 ± 24.1	107.4 ± 20.9	4.5 ± 31.2	0.478
Inferior-temporal	139.4 ± 17.7	143.5 ± 12.8	−4.1 ± 21.7	0.357
**Quadrantile RNFL phase retardation (nm**, ***n*** **=** **24)**				
Superior	70.5 ± 9.6	71.7 ± 6.6	−1.2 ± 11.2	0.604
Inferior	64.8 ± 7.1	65.3 ± 6.5	−0.5 ± 7.9	0.771

P-value is paired t-test; statistical significance threshold α = 0.01. All measurements are mean ± standard deviation.

### Electroretinography

**Figure 4:**
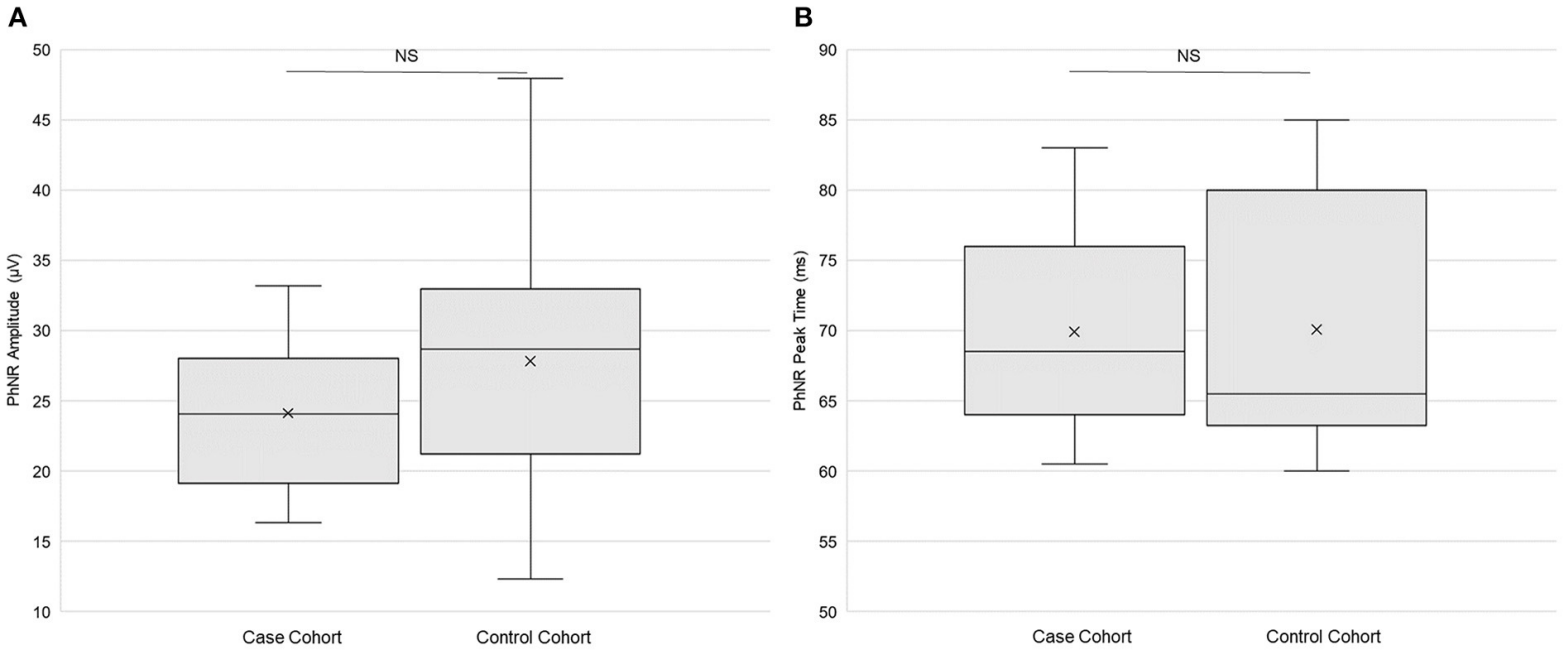
Electroretinography primary outcomes. **(A)** Photopic negative response (PhNR) amplitude (*n* = 20) and **(B)** PhNR peak time (*n* = 21) in both the case and the control cohorts. Each box represents the interquartile range, and the internal line is the median. The internal “X” is the mean. The whiskers represent the 90th and 10th percentiles. Two case subjects (103 and 114) did not return for the ERG study session, and one control subject (204) did not return. PhNR amplitude data from two case subjects (110 and 122) and from one control subject (210) did not meet quality control standards and were thus omitted. PhNR peak time data from one case subject (122) did not meet quality control standards and were thus omitted. NS is not statistically significant (*p* > 0.05, paired *t*-test).

There were no statistically significant differences in PhNR amplitude (*p* = 0.11, paired *t*-test; Figure 4A) or in PhNR peak time (*p* = 0.95; Figure 4B) between the case cohort and the control cohort. There was statistically significantly (*p* < 0.025, *f*-test for equal variances) more variation of PhNR amplitude in the control cohort than in the case cohort (Table 3). Variance in case cohort was not statistically different from variance in the control cohort for PhNR peak time (Table 3). Supplementary Table 2 presents the primary outcomes of ERG testing according to sex.

### Structure-function correlations

For the objective measures of retinal structure and function in the case cohort, there was a weak and not statistically significant negative correlation between global RNFL thickness and PhNR amplitude, between global RNFL thickness and PhNR peak time, and between global RNFL phase retardation and PhNR amplitude (Table 5). There was a moderate negative association between global RNFL phase retardation and PhNR peak time, but this association was not statistically significant (Table 5).

**Table 5:** Associations between retinal nerve fiber layer (RNFL) structural parameters and electroretinography functional parameters in case subjects.

	**Pearson correlation coefficient**	***P*-value**
**Global RNFL thickness, vs**.		
PhNR amplitude (*n* = 21)	−0.15	0.524
PhNR peak time (*n* = 22)	−0.29	0.193
**Global RNFL phase retardation, vs**.		
PhNR amplitude (*n* = 21)	−0.06	0.797
PhNR peak time (*n* = 22)	−0.36	0.098

Statistical significance threshold α = 0.01. PhNR, photopic negative response.

### Traumatic brain injury history

#### Associations between the number of traumatic brain injuries and retinal structure and function

For the primary outcome measures, there was a weak and not statistically significant positive association between number of TBIs and both PhNR amplitude and PhNR peak time (Table 6). Likewise, there was a weak and not statistically significant positive association between number of TBIs and global RNFL thickness (Table 6). There was a weak and not statistically significant negative association between number of TBIs and global RNFL phase retardation (Table 6). Supplementary Figure 1 contains the primary outcome measures both from retinal imaging and from ERG testing for the 14 case subjects with 2–3 TBIs, for the six case subjects with 5–6 TBIs, and for the five case subjects with >5 TBIs.

**Table 6:** Associations between number of traumatic brain injuries (TBIs) and retinal nerve fiber layer (RNFL) structural parameters and electroretinography functional parameters in case subjects.

	**Spearman's rank correlation coefficient**	***P*-value**
**Number of TBIs, vs**.		
Global RNFL thickness (*n* = 25)	0.21	0.32
Global RNFL phase retardation (*n* = 24)	−0.10	0.64
PhNR amplitude (*n* =21)	0.09	0.70
PhNR peak time (*n* = 22)	0.14	0.55

Statistical significance threshold α = 0.01. PhNR, photopic negative response.

For the secondary perimetry metrics, there was a weak (*r* = 0.10, Spearman's Rank) and not statistically significant (*p* = 0.68) positive association between number of TBIs and MD. There was a moderate (*r* = −0.34), but not statistically significant (*p* = 0.15), negative association between number of TBIs and PSD.

#### Associations between time since last traumatic brain injury and retinal structure and function

For the primary outcome measures, there was a weak and not statistically significant negative association between time since last TBI and both global RNFL thickness and global RNFL phase retardation (Table 7). Similarly, there was a very weak and not statistically significant negative association between time since last TBI and PhNR peak time (Table 7). There was a strong positive association between time since last TBI and PhNR amplitude, but this relationship was not statistically significant (Table 7). Supplementary Figure 2 contains the primary outcome measures both from retinal imaging and from ERG testing for the were nine case subjects whose last TBI was <2 years ago, for the nine case subjects whose last TBI was 2–4 years ago, and for the seven case subjects whose last TBI was ≥5 years ago.

**Table 7:** Associations between time since last traumatic brain injury (TBI) and retinal nerve fiber layer (RNFL) structural parameters and electroretinography functional parameters in case subjects.

	**Spearman's rank correlation coefficient**	***P*-value**
**Time since last TBI, vs**.		
Global RNFL thickness (*n* = 25)	−0.16	0.43
Global RNFL phase retardation (*n* = 24)	−0.17	0.43
PhNR amplitude (*n* = 21)	0.53	0.02
PhNR peak time (*n* = 21)	−0.05	0.83

Statistical significance threshold α = 0.01. PhNR, photopic negative response.

For the secondary perimetry metrics, there was a moderate (*r* = 0.32, Spearman's Rank), but not statistically significant (*p* = 0.16), positive association between last TBI and MD. There was also a moderate (*r* = −0.30), but not statistically significant (*p* = 0.20), negative association between last TBI and PSD.

#### Associations between time since first traumatic brain injury and retinal structure and function

For the primary outcome measures, there was a weak and not statistically significant negative association between time since first TBI and global RNFL thickness (Table 8). There was a moderate negative association between time since first TBI and global RNFL phase retardation, and there was a moderate positive association between time since first TBI and PhNR amplitude (Table 8). Neither of these associations were statistically significant. There was a strong positive association between time since first TBI and PhNR peak time, but this relationship was not statistically significant (Table 8). Supplementary Figure 3 contains the primary outcome measures both from retinal imaging and from ERG for the five case subjects whose first TBI was 1–5 years ago, for the eight case subjects whose first TBI was 6–10 years ago, for the eight case subjects whose first TBI was 11–20 years ago, and for the four case subjects whose first TBI was >20 years ago.

**Table 8:** Associations between time since first traumatic brain injury (TBI) and retinal nerve fiber layer (RNFL) structural parameters and electroretinography functional parameters in case subjects.

	**Spearman's rank correlation coefficient**	***P*-value**
**Time since first TBI, vs**.		
Global RNFL thickness (*n* = 25)	−0.21	0.32
Global RNFL phase retardation (*n* = 24)	−0.32	0.13
PhNR amplitude (*n* = 21)	0.45	0.04
PhNR peak time (*n* = 22)	0.50	0.02

Statistical significance threshold α = 0.01. PhNR, photopic negative response.

For the secondary perimetry metrics, there was a moderate (*r* = 0.33, Spearman's Rank), but not statistically significant (*p* = 0.16), positive association between time since first TBI and MD. There was also a moderate (*r* = −0.41), but not statistically significant (*p* = 0.07), negative association between time since first TBI and PSD.

## Discussion

The purpose of this cross-sectional study was to measure the objective structure and the objective function of RGCs in case subjects with a history of repeated TBI and in healthy control subjects. OCT and SLP quantified the thickness and the phase retardation, respectively, of the RNFL. Their global indices were the primary outcome measurements for the structure of the RNFL. There were no statistically significant differences both in global RNFL thickness and in global RNFL phase retardation between the case cohort and control cohort. As a secondary analysis of retinal structure, sectoral RNFL thickness and superior and inferior quadrantile RNFL phase retardation were compared between the two cohorts. Similar to the primary outcome measures, there were no statistical differences between the two cohorts for these secondary outcome measures. Nasal-sector OCT thickness was greater in case subjects than in control subjects, but the difference between the groups was not statistically significant. Studies in Olympic boxers (30) and in United States veterans (32) have reported retinal thickening caused by TBI, which may be attributed to the inflammatory processes that occur in neural tissues after mechanical insult (48).

The negative retinal imaging results of the present study are not unique. In 2015, Capo-Aponte and colleagues reported no statistical difference in global OCT thickness between 17 United States Marines, who had a history of multiple blast exposures, and control subjects without blast exposure (35). Similar to the sample population reported here, the head injuries suffered by the Marines were classified as mild. The results from these two studies suggest that measuring global RNFL thickness may not be a reliable bellwether for the pathology associated with repeated mild TBIs. The lack of statistical difference in global phase retardation between the case cohort and the control cohort in the current study supports this conclusion. SLP measures the phase retardation of polarized light to image the retina. Microtubules in retinal neurons are major contributors to phase retardation (49); thus, SLP may be sensitive to neurodegenerations that compromise microtubules, including repeated TBI. We did not find that, however. Instead, the fact that repeated TBIs did not cause statistically significant changes to RNFL thickness nor to RNFL phase retardation suggests that mild-moderate TBIs may not alter the structure of the RNFL in a general population. Since this is the first investigation of retinal phase retardation in subjects with a history of TBI, more study is needed in this area.

Contrary to our findings, multiple groups report that soldiers (29) and athletes (30, 31, 50), who play contact sports, manifest statistically significant retinal thinning after TBI. The subject population assessed is a key difference between these studies and the present one. Soldiers and especially athletes likely experience more cumulative episodes of head trauma than the general population. For example, athletes who play football experience nearly 1,200 head impacts per year, 12 of which are considered “severe” (51). Case subjects in the current study reported an average of 4.12 lifetime TBIs. Given this disparity in TBI exposures, it is not necessarily surprising that studies conducted exclusively on athletes report RNFL thinning after TBI, but this study on a population with relatively few TBIs caused by a variety of blows to the head did not. Our negative results may be more generalizable to a broad population than studies conducted on soldiers or on athletes. One recent investigation on a general population in India did report RNFL thinning following a TBI (33). This study focused on acute injuries (primary outcome measurements made at 6 months), however, while our study measured people in the chronic stage of TBI (average time since last TBI = 3.8 ± 4.3 years). Such a difference in timeframe might be one reason for disparate results.

The amplitude of and the peak time of the PhNR were measured as objective indicators of RGC function. There were no statistically significant differences in PhNR amplitude or in PhNR peak time between the case cohort and the control cohort. The PhNR is not well-studied in human subjects with TBI, but a mouse model has shown that repeated TBIs elicit a statistically significant reduction in PhNR amplitude (24). Translation of results between animal models of TBI and human subjects is difficult, not only because of substantial differences in anatomy between the species but also because of the varied nature of the injuries experienced by each (52). Human-based studies have employed other methods of assessment to quantify RGC function after TBI. Visual field defects are a commonly reported functional deficit after TBI (53), but they are subjective in nature and present in non-specific patterns (33, 54). Moreover, there were no statistically significant differences in MD or in PDS between the two cohorts of the current study. Alterations to the pupillary light response may provide an objective measure of retinal function in TBI. There is evidence that pupil constriction in response to pulses of blue light is more sustained in subjects with mild TBI than in controls (55), but high constriction variability within TBI subjects may limit the clinical value of this marker (56).

There were no statistically significant differences in variance between the control cohort and the case cohort for global RNFL thickness, for global RNFL phase retardation, and for PhNR peak time; however, there was statistically significantly more variance in the control cohort than in the case cohort for PhNR amplitude. The cause of this variation in controls is unclear, but artifacts such as blinks and eye movements in response to the red-on-blue ERG stimulus may have contributed. Although photophobia is a common symptom after traumatic brain injury (57), TBI subjects do not display more light-adverse reactions to light stimuli than healthy control subjects (58), possibly due to TBI-related damage to frontal-subcortical circuits that results in an inability to produce reflexive behaviors and to simultaneously self-monitor and self-correct them (59). This post-TBI apathy (60) may have dampened the ability of some case subjects to respond to the stimulus with blinks or eye movements, resulting in less variable PhNR amplitude data than non-apathetic control subjects.

Secondary analysis of the PhNR within the case cohort revealed a strong, but not statistically significant, positive association between years since last TBI and PhNR amplitude, and there was a moderate, but not statistically significant, positive association between time since first TBI and PhNR amplitude. Likewise, there was a strong, but not statistically significant, positive association between years since first TBI and PhNR peak time. The association between time since first TBI and PhNR peak time is difficult to interpret, due both to the slow nature of PhNR peak time and to a lack of established assessment criteria for it (42). The strong and moderate, although not statistically significant, associations between time since last TBI and time since first TBI and PhNR amplitude, respectively, may be preliminary evidence for a change in objective RGC function over time after TBI. Our secondary analyses of subjective perimetry metrics may provide additional preliminary evidence for functional changes in RGC function after TBI. There was a moderate, but not statistically significant, association between time since first and last TBIs and improved MD and PSD values. These perimetry results may align with a previous report of recovery of visual field sensitivity after TBI (61).

### Study limitations and future considerations

There are several important limitations to this study. First, the present study was not designed to rigorously investigate the possibility of functional recovery, so definite conclusions cannot be drawn. Future well-powered and longitudinal studies are needed to properly characterize the natural history of PhNR amplitude after TBI. These studies are necessary because, to our knowledge, a change in objectively measured retinal function after TBI has never been demonstrated. Moreover, retinal structure may recover over time after TBI. A longitudinal study on veterans with a history of TBI reported initial retinal swelling, compared to healthy controls, that thinned over 5 years, potentially as neuronal inflammation dissipated (32). ERG testing was not performed as part of the longitudinal study, but visual field sensitivity decreased over time. It is unclear whether this decrease was due to the RNFL thinning or to other factors, such as aging or cataracts.

The second limitation is a small sample size, which may have impaired our ability to detect differences in RGC structure or function between the two cohorts, especially since the effect size of repeated TBIs on the structure and function of RGCs in a general population appears to be small, if present at all. It may also have hindered our ability to establish structure-function relationships and relationships between TBI history and structural and functional parameters in the case subjects.

Third, SLP cannot segment layers within the retina, but instead it measures phase retardation from the entire depth of the retina. As a result, phase retardation changes associated with RNFL pathology may be difficult to differentiate from adjacent retina (62, 63). The combined use of polarized light and cross-sectional imaging in polarization-sensitive OCT can likely overcome these limitations (64–66), but polarization-sensitive OCT is not yet clinically available.

Forth, this study investigated RGCs and did not measure the structure of or the function of other retinal neurons or of the retinal vasculature. The choroid and the outer retina are susceptible to alteration from neurodegenerations (67, 68). Additionally, changes to retinal profusion, as measured by OCT angiography, have been reported after moderate-severe TBI (69). It is unknown, however, if multiple mild-moderate TBIs alter retinal profusion or retinal neurons other than RGCs. Future studies are needed to fill this knowledge gap.

Finally, it was difficult to assure that case subjects really had a TBI and that control subjects did not. We took steps to mitigate this sampling error, however. First, most case subjects were recruited from the OSU binocular vision service, at which they were receiving treatment for visual symptoms following a head injury. Subjects not recruited from the clinic were referred to our study from other TBI researchers at OSU. Second, we used a validated survey to query lifetime TBI history, and we cross-referenced the results of this survey with information contained within the subject's optometry chart, when available. Often, the optometry chart contained correspondence from a physician in neurology or from an occupational or physical therapist, confirming the diagnosis. For control subjects, we also cross-referenced the results of the OSU TBI-ID with an optometry chart to ensure no TBI history.

## Conclusion

In conclusion, this pilot study did not find statistically significant differences in global RNFL thickness, in global RNFL phase retardation, in PhNR amplitude, or in PhNR peak time between a cohort of subjects with a history of repeated TBI and a cohort of healthy control subjects. PhNR amplitude was more variable in control subjects than in case subjects, however. There was a strong, but not statistically significant, association between time since last TBI and PhNR amplitude, and a moderate, but not statistically significant, association between time since first TBI and PhNR amplitude, in case subjects. Future large, longitudinal studies will be necessary to confirm our findings that there is no difference in PhNR amplitude, in PhNR peak time, in RNFL thickness, and in RNFL phase retardation between subjects with a history of multiple TBIs and healthy control subjects. These studies will also be able to more fully investigate the potential interaction between PhNR amplitude and time since first or last TBI.

## Data availability statement

The raw data supporting the conclusions of this article will be made available by the authors, without undue reservation.

## Ethics statement

The studies involving human participants were reviewed and approved by the Institutional Review Board in Biomedical Sciences at The Ohio State University. The patients/participants provided their written informed consent to participate in this study.

## Author contributions

KK and ES-G: subject recruitment, data collection, data processing, data analysis, and manuscript editing. ES and ED: study design, subject recruitment, data collection, and manuscript editing. LJ: study design, data analysis, and manuscript editing. MR: data analysis and manuscript editing. JR and DV: study design, data processing, data analysis, and manuscript editing. CM: study design, subject recruitment, and manuscript editing. PY: research question, study design, subject recruitment, data collection, data processing, data analysis, and manuscript writing. All authors contributed to the article and approved the submitted version.

## Funding

This study was funded by the Lois Hagelberger Huebner Young Investigator Award from the Ohio Lions Eye Research Foundation (PY), the Career Development Award from the American Academy of Optometry (DV), and the NEI T35 EY007151 (KK). PY also received financial support from NEI L30 EY024749.

## Conflict of interest

The authors declare that the research was conducted in the absence of any commercial or financial relationships that could be construed as a potential conflict of interest.

## Publisher's note

All claims expressed in this article are solely those of the authors and do not necessarily represent those of their affiliated organizations, or those of the publisher, the editors and the reviewers. Any product that may be evaluated in this article, or claim that may be made by its manufacturer, is not guaranteed or endorsed by the publisher.
