# Supplementary material for: Structure and function of retinal ganglion cells in subjects with a history of repeated traumatic brain injury
Source: Front Neurol. 2022 Aug 12;13:963587. doi: 10.3389/fneur.2022.963587 (PMC9412167; doi:10.3389/fneur.2022.963587)
Supplement: Supplementary file 1 [file Data_Sheet_1.DOCX]

Supplementary Material

# Supplementary Tables

**Table S1**. Primary retinal nerve fiber layer (RNFL) imaging outcomes by sex

|  | Case cohort | Control cohort | Mean difference |
| --- | --- | --- | --- |
| Global RNFL thickness (µm) |  |  |  |
| Male (n = 12) | 95.6 ± 6.7 | 93.9 ± 6.5 | 1.8 ± 9.5 |
| Female (n = 13) | 97.5 ± 11.6 | 95.8 ± 7.7 | 1.7 ± 11.9 |
| Global RNFL phase retardation (nm) |  |  |  |
| Male (n = 11) | 57.5 ± 5.2 | 56.8 ± 4.8 | 0.6 ± 6.1 |
| Female (n = 13) | 58.3 ± 6.2 | 59.4 ± 4.3 | -1.1 ± 6.7 |

All measurements are mean ± standard deviation

**Table S2**. Electroretinography (ERG) outcomes by sex

|  | Case cohort | Control cohort | Mean difference |
| --- | --- | --- | --- |
| PhNR amplitude (µV) |  |  |  |
| Male (n = 8) | 24.8 ± 5.3 | 29.8 ± 12.2 | -5.0 ± 12.0 |
| Female (n = 12) | 23.7 ± 5.2 | 26.5 ± 6.7 | -2.9 ± 8.9 |
| PhNR peak time (ms) |  |  |  |
| Male (n = 8) | 69.2 ± 7.1 | 69.3 ± 9.4 | -0.1 ± 10.3 |
| Female (n = 12) | 71.0 ± 7.0 | 71.3 ± 8.5 | -0.3 ± 13.3 |

All measurements are mean ± standard deviation. PhNR is photopic negative response.

# Supplementary Figures

**
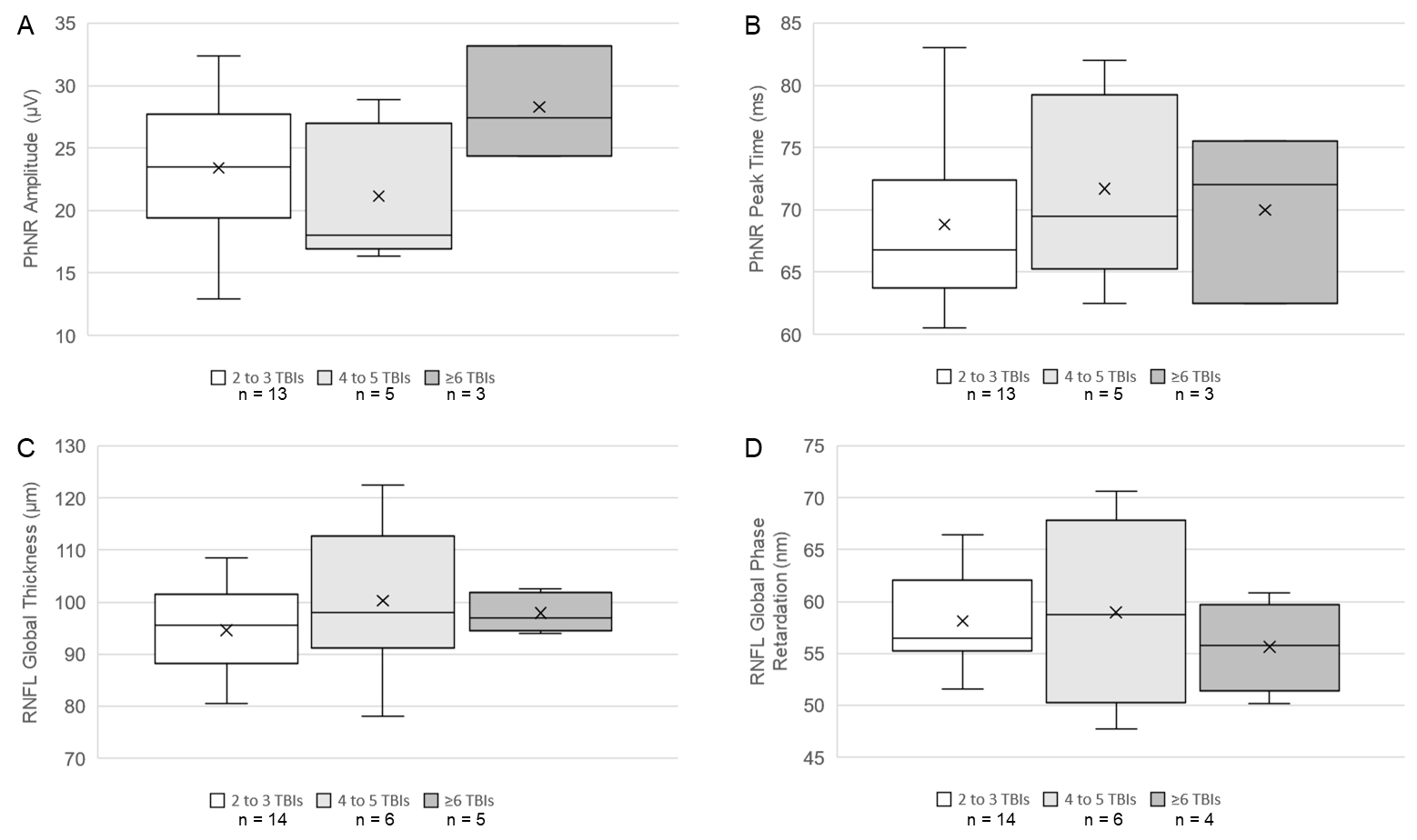
**

**Figure S1**. Primary outcome measures, based on number of lifetime traumatic brain injuries (TBIs). (**A**) Photopic negative response (PhNR) amplitude, (**B**) PhNR peak time, (**C**) retinal nerve fiber layer (RNFL) global thickness, and (**D**) RNFL global phase retardation for case subjects with a lifetime history of 2-3 TBIs, of 4-5 TBIs, or of ≥6 TBIs. Each box represents the interquartile range, and the internal line is the median. The internal ‘X’ is the mean. The whiskers represent the 90th and 10th percentiles.


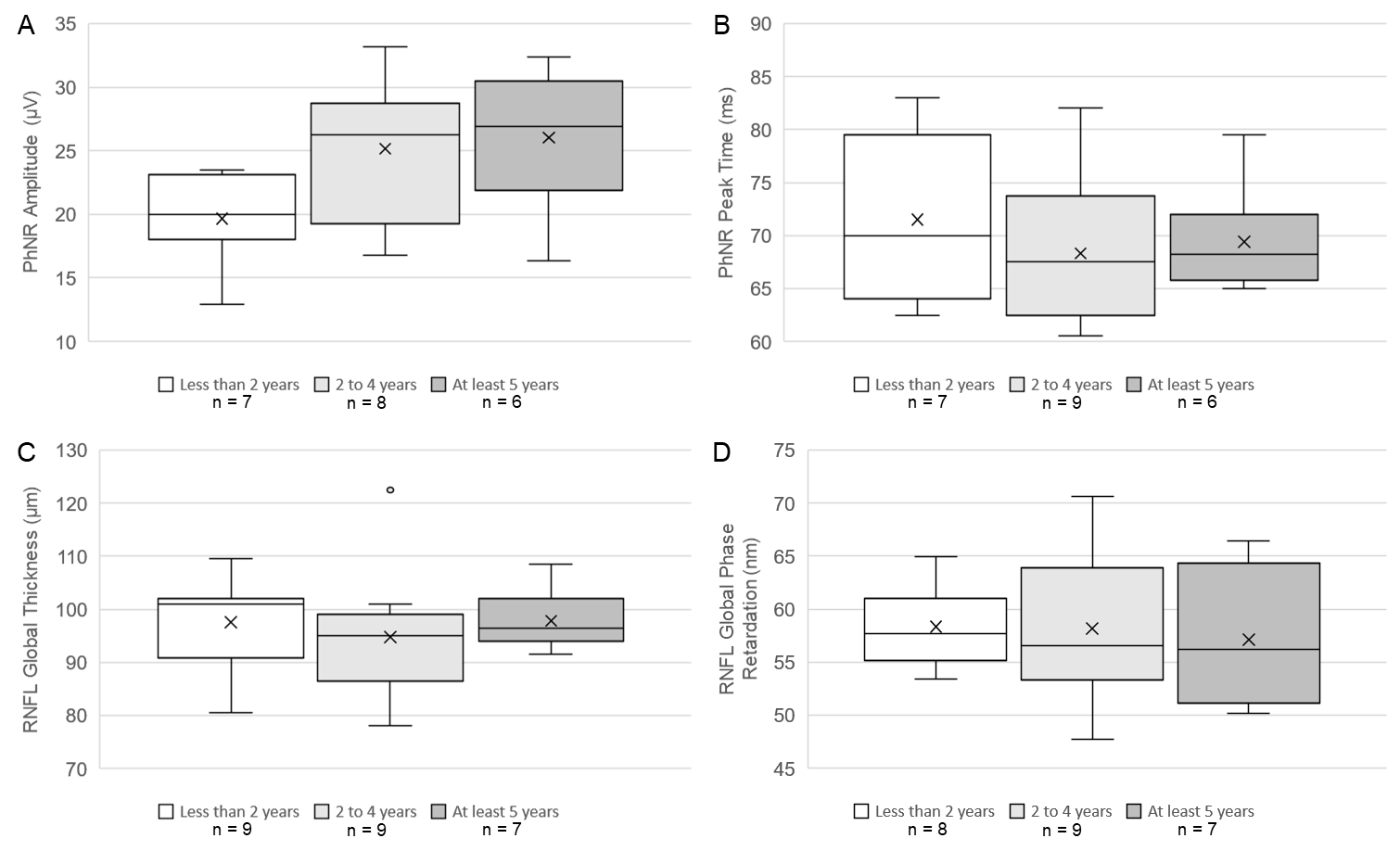


**Figure S2**. Primary outcome measures, based on time since last traumatic brain injury (TBI). (**A**) Photopic negative response (PhNR) amplitude, (**B**) PhNR peak time, (**C**) retinal nerve fiber layer (RNFL) global thickness, and (**D**) RNFL global phase retardation for case subjects ≤2 years since their last TBI, 2-4 years since their last TBI, and ≥5 years since their last TBI. Each box represents the interquartile range, and the internal line is the median. The internal ‘X’ is the mean. The whiskers represent the 90th and 10th percentiles, and the filled circle is an outlying value.

**
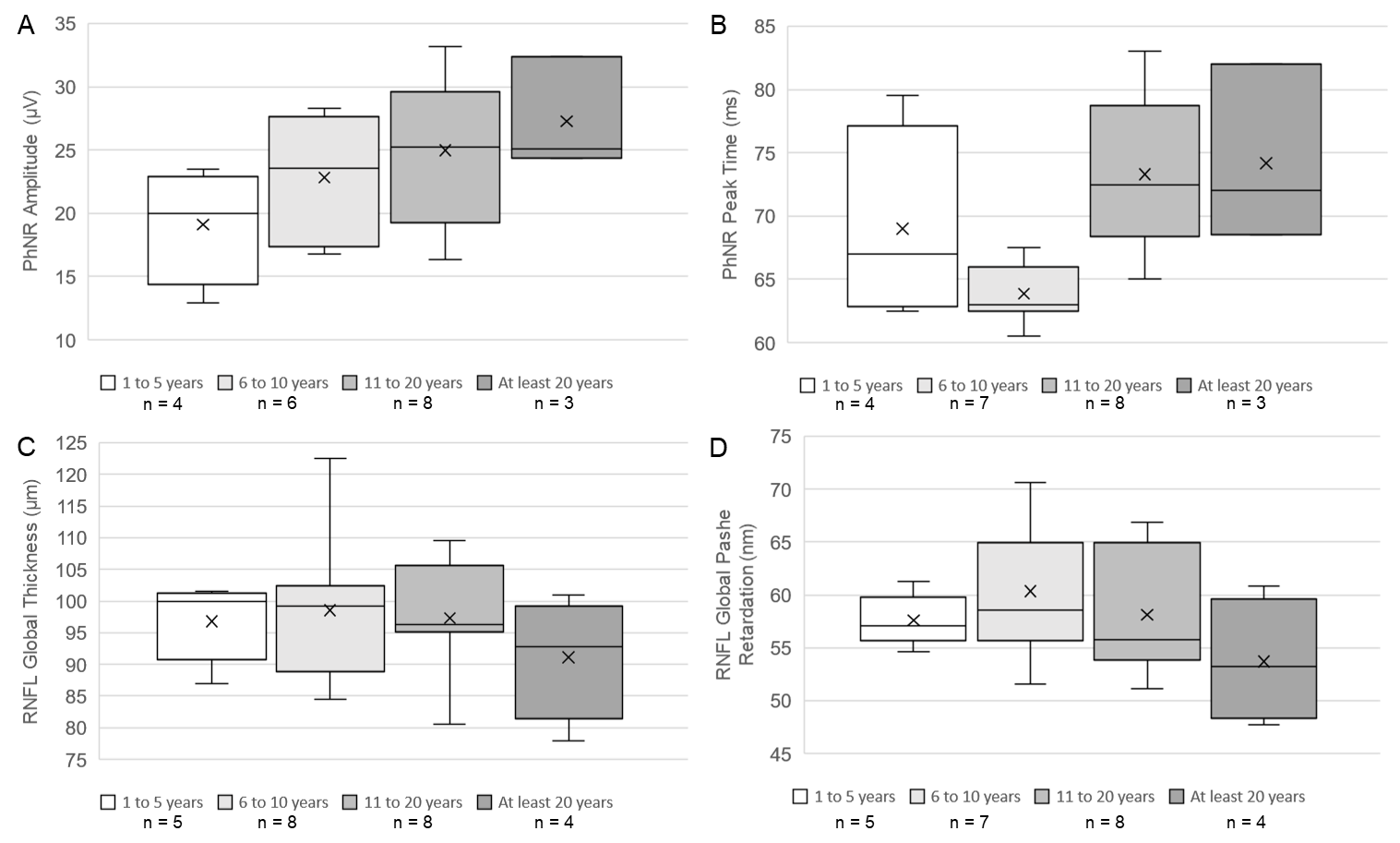
**

**Figure S3**. Primary outcome measures, based on time since first traumatic brain injury (TBI). (**A**) Photopic negative response (PhNR) amplitude, (**B**) PhNR peak time, (**C**) retinal nerve fiber layer (RNFL) global thickness, and (**D**) RNFL global phase retardation for case subjects 1-5 years since their first TBI, 6-10 years since their first TBI, 11-20 years since their first TBI, and ≥20 years since their first TBI. Each box represents the interquartile range, and the internal line is the median. The internal ‘X’ is the mean. The whiskers represent the 90th and 10th percentiles.
